# Structure/Function Studies of the α4 Subunit Reveal Evolutionary Loss of a GlyR Subtype Involved in Startle and Escape Responses

**DOI:** 10.3389/fnmol.2018.00023

**Published:** 2018-01-31

**Authors:** Sophie Leacock, Parnayan Syed, Victoria M. James, Anna Bode, Koichi Kawakami, Angelo Keramidas, Maximiliano Suster, Joseph W. Lynch, Robert J. Harvey

**Affiliations:** ^1^Department of Pharmacology, UCL School of Pharmacy, London, United Kingdom; ^2^Queensland Brain Institute, The University of Queensland, Brisbane, QLD, Australia; ^3^Division of Molecular and Developmental Biology, National Institute of Genetics and Department of Genetics, Graduate University for Advanced Studies (SOKENDAI), Mishima, Japan; ^4^Neural Circuits and Behaviour Group, Uni Research AS, Bergen, Norway; ^5^School of Biomedical Sciences, The University of Queensland, Brisbane, QLD, Australia; ^6^School of Health and Sport Sciences, University of the Sunshine Coast, Sippy Downs, QLD, Australia; ^7^Sunshine Coast Health Institute, Birtinya, QLD, Australia

**Keywords:** α4 subunit, glycine receptor, *GLRA4*, hyperekplexia, startle disease, zebrafish

## Abstract

Inhibitory glycine receptors (GlyRs) are pentameric ligand-gated anion channels with major roles in startle disease/hyperekplexia (GlyR α1), cortical neuronal migration/autism spectrum disorder (GlyR α2), and inflammatory pain sensitization/rhythmic breathing (GlyR α3). However, the role of the GlyR α4 subunit has remained enigmatic, because the corresponding human gene (*GLRA4*) is thought to be a pseudogene due to an in-frame stop codon at position 390 within the fourth membrane-spanning domain (M4). Despite this, a recent genetic study has implicated *GLRA4* in intellectual disability, behavioral problems and craniofacial anomalies. Analyzing data from sequenced genomes, we found that GlyR α4 subunit genes are predicted to be intact and functional in the majority of vertebrate species—with the exception of humans. Cloning of human GlyR α4 cDNAs excluded alternative splicing and RNA editing as mechanisms for restoring a full-length GlyR α4 subunit. Moreover, artificial restoration of the missing conserved arginine (R390) in the human cDNA was not sufficient to restore GlyR α4 function. Further bioinformatic and mutagenesis analysis revealed an additional damaging substitution at K59 that ablates human GlyR α4 function, which is not present in other vertebrate GlyR α4 sequences. The substitutions K59 and X390 were also present in the genome of an ancient Denisovan individual, indicating that *GLRA4* has been a pseudogene for at least 30,000–50,000 years. In artificial synapses, we found that both mouse and gorilla α4β GlyRs mediate synaptic currents with unusually slow decay kinetics. Lastly, to gain insights into the biological role of GlyR α4 function, we studied the duplicated genes *glra4a* and *glra4b* in zebrafish. While *glra4b* expression is restricted to the retina, using a novel *tol2*-GAL4FF gene trap line (SAIGFF16B), we found that the zebrafish GlyR α4a subunit gene (*glra4a*) is strongly expressed in spinal cord and hindbrain commissural neurones. Using gene knockdown and a dominant-negative GlyR α4a^R278Q^ mutant, we found that GlyR α4a contributes to touch-evoked escape behaviors in zebrafish. Thus, although GlyR α4 is unlikely to be involved in human startle responses or disease states, this subtype may contribute to escape behaviors in other organisms.

## Introduction

Inhibitory glycine receptors (GlyRs) are ligand-gated anion channels, consisting of pentameric combinations of GlyR α and β subunits. There are five known GlyR subtypes (containing α1, α2, α3 or α4 subunits together with the GlyR β subunit) that are differentially expressed in developing brain and adult spinal cord, hindbrain, cerebellum and retina. Mutations in *GLRA1* and *GLRB*, encoding the GlyR α1 and β subunits, cause startle disease/hyperekplexia, a neurological disorder characterized by noise- or touch-induced seizures in neonates (Shiang et al., 1993; Rees et al., 2002; Chung et al., 2010, 2013; James et al., 2013). Allelic variants of *GLRB* may also contribute to the risk of panic disorder by increasing startle responses and thus agoraphobic cognitions (Deckert et al., 2017). However, the biological roles of other GlyR subtypes, containing the α2, α3 and α4 subunits, are still under investigation. Knockout mice have revealed roles for GlyR α2 in retinal rod photoreceptor development, crossover inhibition and the receptive field surround of “off” retinal ganglion cells (Young and Cepko, 2004; Nobles et al., 2012; Zhang C. et al., 2015). GlyR α2 also appears to modulate ethanol consumption, since knockout mice show reduced ethanol intake and preference in the two-bottle choice test (Blednov et al., 2015). Most recently, GlyR α2 has been shown to control cortical neuronal progenitor homeostasis, migration and circuit formation (Avila et al., 2013, 2014; Morelli et al., 2017) with mild microcephaly (Avila et al., 2014), susceptibility to seizures (Morelli et al., 2017) and deficits in long-term potentiation and object recognition memory (Pilorge et al., 2016) observed in *Glra2* knockout mice. Consistent with these findings, loss of function mutations in the human GlyR α2 subunit gene (*GLRA2*) have been reported in cases of autism spectrum disorder, with additional features in some cases such as language delay and seizures (Pinto et al., 2010; Piton et al., 2011; Iossifov et al., 2014; Pilorge et al., 2016). By contrast, the generation of GlyR α3 subunit knockout mice and subtype specific antibodies revealed that this subtype is abundant in the spinal cord dorsal horn, where it plays a key role in central inflammatory pain sensitization (Harvey et al., 2004, 2009; Hösl et al., 2006). This has led to significant interest in GlyR α3 as a target for novel analgesics (Harvey et al., 2004; Xiong et al., 2011; Balansa et al., 2013; Han et al., 2013; Acuña et al., 2016; Stead et al., 2016; Huang et al., 2017). However, it is important to note that GlyR α3 also has other biological roles. For example, a 5-HTR_1A_-GlyR α3 signaling pathway controls rhythmic breathing in the brainstem pre-Bötzinger complex (Manzke et al., 2010), with disruption of this pathway in GlyR α3 knockout mice resulting in an irregular respiratory rhythm. GlyR α3 knockout mice also show increased ethanol intake, preference and increased development of conditioned taste aversion to ethanol (Blednov et al., 2015). Lastly, GlyR α3 is involved in hearing, and has important functions in auditory nerve activity (Dlugaiczyk et al., 2016) and signal-in-noise detection (Tziridis et al., 2017).

By comparison, the GlyR α4 subtype is poorly studied, largely because the human gene (*GLRA4*) is considered to be a pseudogene (Simon et al., 2004) due to the presence of an in-frame stop codon at position 390 (390X) in exon 9, truncating the GlyR subunit within the fourth membrane-spanning domain (M4). This often leads to the incorrect assumption that *GLRA4* is not expressed in human brain (Bar-Shira et al., 2015) and is therefore not biologically relevant. However, one mystery that remains unexplained is why the GlyR α4 subunit gene appears to be intact in all other species studied to date (Matzenbach et al., 1994; Harvey et al., 2000) and is even duplicated (GlyR α4a and α4b) in zebrafish (Imboden et al., 2001; Hirata et al., 2010). GlyR α4 subunit expression has been detected by *in situ* hybridization and PCR assays in chicken embryonic sympathetic neurons, where depolarizing GlyRs have been linked to neurotransmitter release (Boehm et al., 1997; Harvey et al., 2000). GlyR α4 was also found in spinal cord white matter, dorsal root ganglia and the male genital ridge in birds (Harvey et al., 2000). More recently, the development of new subunit-specific antibodies also allowed localization of GlyR α4 in cholinergic amacrine cells in mouse retina (Heinze et al., 2007). Interest in GlyR α4 has recently been rekindled by reports that *GLRA4* in humans is potentially involved in intellectual disability, behavioral problems and craniofacial anomalies (Labonne et al., 2016). An 11-year-old female patient (DGDP084) with these symptoms was reported to have a *de novo* Xq22.2 110 kb microdeletion encompassing *GLRA4*, *MORF4L2* and *TCEAL*. While certain phenotypic features such as cognitive impairment and motor delay overlap with Pelizaeus-Merzbacher disease (PMD) caused by *PLP1* mutations at Xq22.2, this gene was apparently not included in the microdeletion and was not dysregulated by a positional effect (Labonne et al., 2016). Since GlyR α4 transcripts were reduced in the female patient compared to her healthy mother, the authors suggested that loss of one allele of *GLRA4* was a plausible explanation for the clinical symptoms observed in this individual. However, in this study, we demonstrate that although the human GlyR α4 subunit gene is expressed and correctly spliced, multiple inactivating substitutions render the human GlyR α4 subunit dysfunctional in modern and ancient humans. We also investigate the physiological properties of inhibitory synaptic currents mediated by α4β GlyRs in artificial synapses. Finally, we report that the expression pattern and knockdown of the zebrafish GlyR α4a subunit are consistent with a role for the GlyR α4 subunit in mediating startle and escape responses.

## Materials and Methods

### Phylogenetic Analysis and Cloning of GlyR α4 Subunit Sequences

The human GlyR α4 subunit was aligned with orthologs predicted from Denisova, Neanderthal and other vertebrate genomes obtained via Ensembl version 87 (Aken et al., 2017) or the UCSC Genome Browser (Kent et al., 2002). Alignments were made and edited using CLC Main Workbench 6 software. Positions of membrane-spanning domains and other structural features were mapped based on the recent cryo-EM structures of the zebrafish GlyR α1 subunit (3JAE; Du et al., 2015). Human and mouse GlyR α4 subunit cDNAs were amplified from whole-brain first-strand cDNA (Clontech, Cat. 637242 and 637301) using primers hGlyR α4-BamHI 5′-caaggatccgccaccatgacaactcttgttcctgc-3′/hGlyR α4-XhoI 5′-ccactcgagtcacagagcctggtggatatc-3′ or mGlyR α4-EcoRI 5′-caagaattcgccaccatgacaactcttgttccagcaa-3′/mGlyR α4-SalI 5′- ccagtcgactcacagtgcctggtggatatctt-3′, cloned into the expression vector pRK5. Gorilla and Chimp GlyR α4 subunit cDNAs were artificially synthesized from predicted cDNA sequences (Gorilla: XM_004064625.1; Chimp: XM_009439421.2) and recloned into pRK5 using the primers gGlyR α4EcoRI 5′-caagaattcgccaccatgacaactcttgttcctgaaa-3′/gGlyR α4SalI and 5′-ccagtcgactcacagtgcctggtggatatctt-3′ and cGlyR α4-EcoRI 5′-caagaattcgccaccatgacaactcttgttcctgcaa-3′/cGlyR α4-SalI 5′-cttgtcgactcacagagcctggtggatatctt-3′, respectively. In each case, an optimized Kozak sequence was introduced upstream of the start methionine (GCCACC in oligonucleotide sequences).

### Site-Directed Mutagenesis and DNA Sequencing

Sequence changes were introduced into pRK5-human GlyR α4 and pRK5-mouse GlyR α4 using the QuikChange Lightning site-directed mutagenesis kit (Agilent). All expression constructs were confirmed by Sanger DNA sequencing of the entire coding region and analyzed using Sequencher 4.10 (Gene Codes Corporation). Sanger DNA sequencing was performed by DNA Sequencing and Services (MRCPPU, College of Life Sciences, University of Dundee, Scotland).

### Primary Culture of Spinal Neurons

Spinal neurons were prepared using methods as recently described (Dixon et al., 2015). Briefly, E15 timed-pregnant rats were euthanized via CO_2_ inhalation in accordance with procedures approved by the University of Queensland Animal Ethics Committee. The spinal cords were rapidly removed, triturated and plated onto poly-D-lysine-coated coverslips in a 4-well plate at a density of 8–10 × 10^4^ cells/well, and cultured for 3–4 weeks until spontaneous inhibitory postsynaptic currents (IPSCs) could be detected. The cells were initially cultured in Dulbecco’s Modified Eagles Medium (DMEM) supplemented with 10% fetal bovine serum (DMEM-FBS). After 24 h the entire DMEM-FBS medium was replaced with Neurobasal medium including 2% B27 and 1% GlutaMAX supplements. A second (and final) feed 1 week later replaced half of this medium with fresh Neurobasal medium. Neurons were used in co-culture experiments between 1–4 weeks later.

### HEK293 Cell Culture, Transfection and Artificial Synapse Formation

Artificial synapses were generated as previously described (Zhang Y. et al., 2015). Briefly, HEK293 cells were cultured in DMEM-FBS until ~90% confluent. One day prior to transfection, they were trypsinized and plated onto glass coverslips in 35 mm culture dishes at a density of 5 × 10^3^ cells/dish. Homomeric channels were transfected with 0.3 μg of α4 subunit constructs (pRK5). Heteromeric channels were transfected at a ratio of 1:50, with 0.02 μg GlyR α4 and 1 μg β subunit constructs (pRK5). 0.1 μg EGFP (pEGFP) was used as a transfection marker. For artificial synapses, 0.3 μg of mouse neuroligin 2A (pNice) and 0.3 μg of rat gephyrin (pCIS) were also added. Transfection was performed via a Ca^2+^ phosphate-DNA co-precipitation method for 15–20 h in a 3% CO_2_ incubator and terminated by washing cells twice with divalent cation-free phosphate buffered saline. Cells were trypsinized the next day, centrifuged and re-suspended in Neurobasal medium (including 2% B27 and 1% GlutaMAX supplements) then seeded onto the neurons. One 35 mm dish of HEK293 cells was typically sufficient to seed four coverslips of neurons. Once seeded with HEK293 cells, the co-cultures were returned to the incubator overnight to allow artificial synapses to form between neurons and transfected HEK293 cells. Cells were used for patch-clamp recording over the following 2–3 days.

### Electrophysiology

Whole-cell patch clamp recordings were performed at room temperature (22 ± 1°C). Glycine concentration-response relationships were performed at −40 mV, whereas artificial synapse recordings were performed at −70 mV, both using a Multiclamp 700B amplifier and pClamp10 software (Molecular Devices). Signals were filtered at 4 kHz and sampled at 10 kHz. Patch pipettes (4–8 MΩ resistance) were fabricated from borosilicate glass (GC150F-7.5, Harvard Apparatus) and filled with an internal solution comprising (in mM): 145 CsCl, 2 CaCl_2_, 2 MgCl_2_, 10 HEPES and 10 EGTA, adjusted to pH 7.4 with CsOH. The extracellular solution comprised (in mM) 140 NaCl, 5 KCl, 2 CaCl_2_, 1 MgCl_2_, 10 HEPES and 10 D-glucose, adjusted to pH 7.4 with NaOH.

Outside-out macropatch recordings were performed at −70 mV using an Axopatch 200B amplifier, pClamp 10 software, filtered at 10 kHz and sampled at 50 kHz. Current traces were filtered off-line at 5 kHz for making figures. Pipettes were fire-polished to a resistance of ~10 MΩ and filled with the same internal solution. Outside-out patches pulled from transfected HEK293 cells were activated by brief (<1 ms) exposure to glycine using a piezo-electric translator (Siskiyou). The speed of the solution exchange system was regularly calibrated by rapidly switching the solution perfusing an open patch pipette between standard extracellular solution and an extracellular solution that had been diluted by 50% with distilled water. By monitoring the resulting pipette current, we were able to ensure that the solution perfusing the macropatch was completely exchanged within 200 μs (Dixon et al., 2014).

### Data Analysis

Analyses of IPSC amplitudes, 10%–90% rise times, and decay time constants were performed using Axograph X (Axograph Scientific). Only cells with a stable series resistance of <25 MΩ throughout the recording period were selected for analysis. IPSCs were detected using a semi-automated sliding template. Each detected event was visually inspected and only those with no inflections in the rising or decay phases were included. All selected events from a single cell were digitally averaged. Parameters derived from these digitally averaged waveforms were then pooled with those form other cells to obtain group data. To calculate macroscopic current decay time constants, digitally averaged macroscopic recordings were fitted with double-exponential functions in Axograph X, and a weighted time constant was calculated from individual time constants (τ1, τ2) and their relative amplitude (A1, A2) as follows: τ_weighted_ = (τ1 × A1 + τ2 × A2)/(A1 + A2).

Displayed averaged data represent group means ± SEMs. The Hill equation was used to calculate the saturating current magnitude (*I*_max_), half-maximal concentration (EC_50_) and Hill coefficient (*n*_H_) values for glycine activation. Individual concentration-response relationships were fitted using a nonlinear least squares algorithm (SigmaPlot 11.0; Jandel Scientific, San Rafael, CA, USA). Statistical analysis and graphing were performed with SigmaPlot 11.0. Group data were tested for normal probability distribution and for significant differences between groups using one-way ANOVA. Pair-wise comparisons were determined using Dunnett’s *post hoc* test, where *p* < 0.05 was taken as the significance threshold.

### Fluorescence-Based Imaging

Cells were imaged using an automated fluorescence-based screening system using EYFP^I152L^ fluorescence quench as an indicator of anion influx. In this technique, iodide flowing into the cell binds to and quenches EYFP^I152L^ fluorescence, thus providing an indication of the relative activity levels of membrane anion channels (Kruger et al., 2005; Gilbert et al., 2009). Briefly, HEK293 cells were transfected with the plasmid DNAs for wild-type and mutant GlyR α4 constructs together with a pEYFP^I152L^ expression construct and plated into a 384-well plate. Unless otherwise indicated, all GlyR plasmid DNAs were transfected in equimolar ratios. Within the following 24–32 h, the culture medium in the wells was replaced with extracellular solution (140 mM NaCl, 5 mM KCl, 2 mM CaCl_2_, 1 mM MgCl_2_, 10 mM HEPES, and 10 mM glucose, pH 7.4 using NaOH). After 30 min, fluorescence images of each well were obtained twice, before and after the application of NaI solution (140 mM NaI, 5 mM KCl, 2 mM CaCl_2_, 1 mM MgCl_2_, 10 mM HEPES and 10 mM glucose, pH 7.4 using NaOH) containing varying concentrations of glycine. Values were pooled from three to four experiments with three wells each containing >200 cells. To determine the glycine dose-response curve, an empirical three or four parameter Hill equation was fitted by a non-linear least squares algorithm using SigmaPlot 11.0 software. Throughout this study, “% quench” is defined as the (initial fluorescence − final fluorescence) × 100/initial fluorescence. Thus, a treatment that completely abolished all fluorescence would yield a 100% quench.

### Immunolabeling and Imaging

Briefly, neuron-HEK293 cell co-cultures were fixed in 4% paraformaldehyde for 15 min, washed with PBS and then incubated in a blocking solution, containing 1% bovine serum albumin in PBS, to minimize the background fluorescence caused by non-specific antibody binding. The antibody-containing solutions were diluted in the same blocking solution. The cultures were first incubated overnight at room temperature with GlyR-specific mouse monoclonal mAb4a (Synaptic Systems, Germany), diluted 1:6250. After washing with PBS, the cultures were incubated for 3 h with 1:1000 donkey anti-mouse antibody and labeled with Alexa-555 (ThermoFisher, Australia). After washing thoroughly with PBS, cultures were incubated in a 1:6250 dilution of mouse monoclonal primary antibody against the presynaptic protein, synaptotagmin 1, labeled with Oyster 650 fluorophore (Synaptic Systems, Germany). After overnight incubation, cultures were washed with PBS, and coverslips mounted onto glass slides using DABCO mounting solution prepared as described (Johnson et al., 1982), sealed with acrylic and stored at 4°C.

### Animal Care

Zebrafish were maintained and used for experiments in accordance with the Norwegian Animal Protection Act and with approval from Mattilsynet (the Norwegian Food Safety Authority). Adults were reared at a maximal density of five animals per liter at 28.5°C in a 14/10 (light/dark) cycle environment. Fish were fed a mixture of live artemia and TetraMin fish flakes twice a day. Larvae were raised at 28.5°C with a 14/10 day/night light cycle. All experiments were performed at room temperature on 1–3 days post fertilization (dpf) larvae unless stated otherwise.

### Zebrafish GlyR α4a Gene Trap Line

The zebrafish SAIGFF16B line was generated by the method described in Kawakami et al. (2010) in which gene trap vectors based on the *Tol2* transposable element in combination with the Gal4FF-UAS system were used. The gene trap construct T2KSAIGFF contains the rabbit β-globin splice acceptor (SA), followed by an internal ribosome entry site (IRES), the coding region for Gal4FF (a modified version of the yeast Gal4 transcription activator) and a downstream polyadenylation site (pA). In zebrafish line SAIGFF16B, this cassette is inserted between exons 1 and 2 of *glra4a* on zebrafish chromosome 14^1^. Gal4FF expression in SAIGFF16B was visualized by creating double transgenic fish carrying a Gal4FF transgene and the GFP reporter gene placed downstream of the Gal4-recognition sequence (UAS:GFP) as previously described.

### Zebrafish GlyR α4a Knockdown and α4a^R278Q^ Mutant Expression

Specific antisense translation-blocking and splice-blocking morpholinos (MOs) for GlyR α4a morpholinos (MOs) were designed using alignments of exon 1 and exon 7 of zebrafish GlyR genes and synthesized by GeneTools LLC. The morpholino sequences used were: *glra4a*SMO1 5′-acctagaagagcacaaagagtttca-3′, *glra4a*SMO2 5′-acaggaactcattttatgttacctt-3′, *glra4a*TBMO 5′-aaatccttatgacctgagggagcat-3′. We determined the optimal MO amount required for induction of a specific phenotype post-injection without inducing toxicity and off-target effects. *Glra4a*TBMO was injected at 2 mM, *glra4a*SMO1 and *glra4a*SMO2 were injected at 1 mM. RT-PCR was also used to examine splicing defects caused by *glra4a*SMO1 and *glra4a*SMO2 using poly(A) + RNA isolated at 72 hours post fertilization (hpf) using primers *glra4a*Ex6F 5′-GCGGATGACTTGACTCTTCCTCAG-3′ and *glra4a*Ex8R 5′-CCTTGAGACGAAGTTGACTGCTGCGTACTC-3′ using primers for *glra1* as controls: *glra1*Ex6F 5′-CTGAC GTTACCTCAGTTTATATTG-3′ and *glra1*Ex8R 5′-GCG CAGAAGCTCCTTGTGTTGGCG-3′. A dominant-negative mutation p.R278Q in was introduced into a zebrafish GlyR α4a subunit cDNA cloned into the pCS2 expression vector using the QuikChange site directed mutagenesis technique, with primers R278Q1 5′-ccacccagagctccggttcacaagcctcgctac-3′ and R278Q2 5′-gtagcgaggcttgtgaaccggagctctgggtgg-3′. *In vitro* transcribed RNA for this construct was made with SP6 RNA polymerase and microinjected into zebrafish embryos at a final concentration of 250 ng/ml.

## Results

### *GLRA4* Is a Pseudogene in the Human Lineage, but Intact in Other Primates

**Figure 1:**
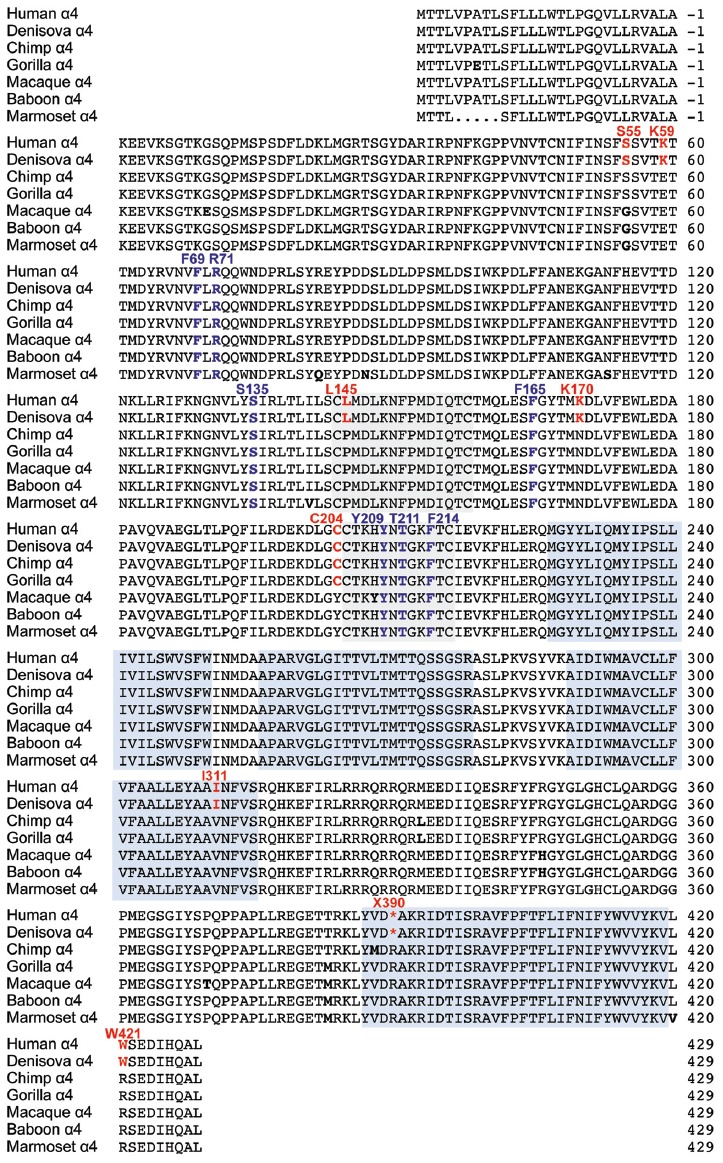
Comparison of glycine receptor (GlyR) α4 subunit sequences in modern and ancient humans and selected primates. The position of the N-terminal signal peptide is indicated by negative numbering, and potential cys-cys loops and membrane-spanning domains (M1–M4) are denoted by gray and blue boxes, respectively. Residues in bold indicate key differences in aligned sequences, while those indicated in red type denote key changes in the human and Denisovan GlyR α4 subunits compared with other primates (although note C204 is also found in gorilla and chimp GlyR α4). Residues in purple type indicate key determinants of the GlyR agonist binding site.

**Figure 2:**
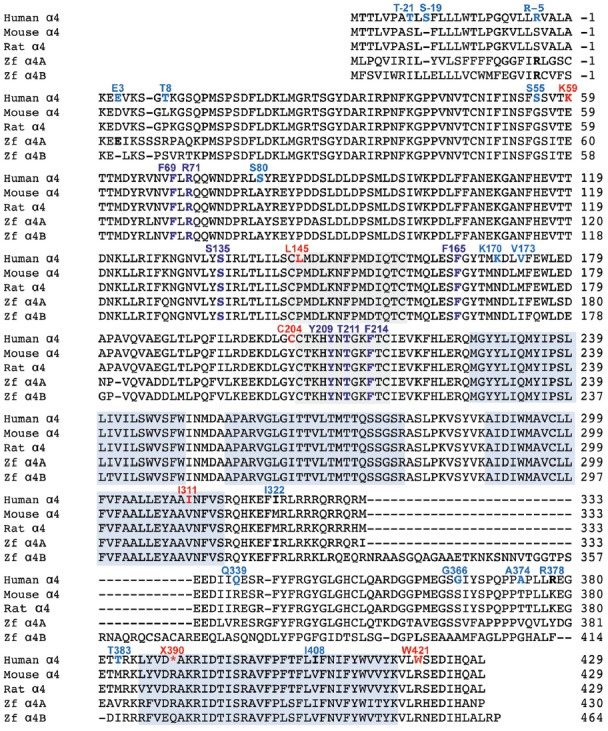
Comparison of GlyR α4 subunit sequences in humans, rodents and zebrafish. The position of the N-terminal signal peptide is indicated by negative numbering, and potential cys-cys loops and membrane-spanning domains (M1–M4) are denoted by gray and blue boxes, respectively. Residues in bold indicate key differences in aligned sequences, while those indicated in blue and red type denote predicted non-damaging (blue) and damaging (red) changes in the human GlyR α4 subunit compared with rodent GlyR α4 subunits predicted by bioinformatics analysis (Table 1). Residues in purple type indicate key determinants of the GlyR agonist binding site.

To confirm that the human GlyR α4 subunit gene (*GLRA4*) is transcribed and correctly spliced, we amplified full-length cDNAs from human hippocampal and whole-brain first-strand cDNA (Clontech) using proofreading *Pfx* DNA polymerase. Sanger DNA sequencing of 10 human GlyR α4 subunit cDNAs and comparison with the *GLRA4* consensus sequence (hg38) revealed that: (1) All cDNAs encoded a valine at position 57 (the more common variant of a known SNV; rs4907817, C: 97.005%; T: 2.995%); (2) Two cDNA clones had the change c.1345T >C, p.W421R, suggesting that this could be a common polymorphism in human *GLRA4*. Importantly, all ten cDNA clones contained an in-frame stop codon (TGA) at position 390 in exon 9, truncating the GlyR α4 subunit prematurely within the fourth membrane spanning domain (M4). Despite this interruption, the coding region continues intact, encoding the rest of M4 and a C-terminus with high sequence identity to the corresponding sequence in other species (Figures 1, 2). We found no evidence of RNA editing of this stop codon, nor alternative splicing of *GLRA4* transcripts that might restore a full reading frame. We also analyzed the sequence of *GLRA4* in a high-coverage genome sequence of a Denisovan, an extinct relative of Neanderthals (Reich et al., 2010). This analysis indicated the sequence of the Denisova GlyR α4 subunit is identical to that of modern day humans, including polymorphic reads at p.W421R, suggesting that *GLRA4* has been a pseudogene for at least 30,000–50,000 years.

The human GlyR α4 subunit consensus sequence was aligned with equivalent proteins from primates and other vertebrates, predicted from sequenced genomes accessed via Ensembl release 87. Two alignments are shown in Figures 1, 2—an alignment of human GlyR α4 subunit with orthologs from ancient humans (Denisova) and different ape species, including chimpanzee, gorilla, macaque, baboon and marmoset (Figure 1) and human GlyR α4 subunit aligned with the mouse, rat and zebrafish GlyR α4 subunits (Figure 2). This analysis shows that GlyR α4 subunit sequences are most divergent in the N-terminal signal peptide and the intracellular loop between transmembrane domains M3 and M4. Outside these regions, key differences between the human and Denisovan GlyR α4 subunit and orthologs from ape species include: (1) Extracellular domain: S55, K59, L145, K170, C204 (although note that S55 and C204 are also found in chimpanzee and gorilla sequences); (2) Transmembrane domain M3: I311; and (3) C-terminal extracellular loop: W421. Notably, the stop codon at position 390 is not present in any of the ape, rodent or fish species, where either an arginine or glutamine is found.

### Functional Analysis of Recombinant α4 GlyRs in HEK293 Cells

**Figure 3:**
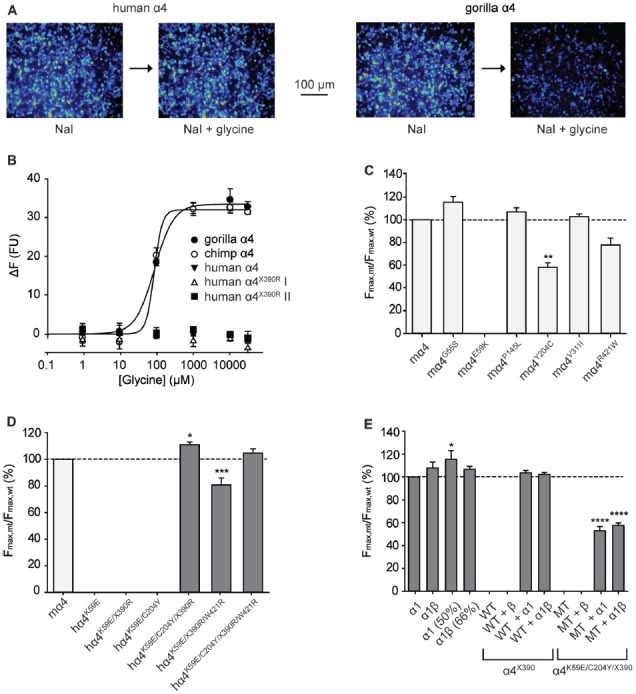
Functional analysis of α4 GlyRs using a fluorescence-based anion influx assay. **(A)** Sample images from HEK293 cells expressing YFP plus the human α4 GlyR (left panel) or the gorilla α4 GlyR (right panel). Images were recorded in the presence of NaI bathing solution before and after a 10 s application of 10 mM glycine. **(B)** Glycine concentration-response results for gorilla, chimpanzee and human GlyR α4 subunits. Two independent full-length human GlyR α4 subunit expression constructs each containing the R390X stop codon were tested. The fluorescence change is plotted against the applied glycine concentration in micromolar. All displayed data points represent the average quench from three experiments with three wells each and >200 cells per well. **(C)** Normalized maximal changes in fluorescence observed upon the addition of NaI containing saturating (10 mM) glycine for the indicated potentially damaging mouse GlyR α4 mutants. The maximal change in fluorescence is presented as the final (quenched) fluorescence value minus the initial fluorescence value. **(D)** Normalized maximal changes in fluorescence observed upon the addition of NaI containing saturating glycine for the indicated potentially rescued human GlyR α4 subunit receptors. Mouse GlyR α4 subunit represented in light gray, human GlyR α4 subunit mutants in dark gray. **(E)** Normalized maximal changes in fluorescence observed upon the addition of NaI containing saturating glycine for wild-type human GlyR α4^X390^ subunits and human GlyR α4^X390/K59E/C204Y^ mutant receptors. GlyR α1 (50%) means that only half the amount of GlyR α1 was transfected, which represents the amount of GlyR α1 when the GlyR α4 subunit was co-transfected with α1. GlyR α1β (66%) represents the corresponding heteromeric state. WT, wild-type GlyR α4^X390^; MT, mutant GlyR α4^K59E/C204Y/ X390^. In **(C–E)**, mutant values were normalized relative to the wild-type value obtained from the same plate. *p*-values were calculated relative to mouse GlyR α4 and the human GlyR α1 using one-way ANOVA followed by Dunnett’s *post hoc* test: *p < 0.05, **p < 0.01, ***p < 0.001, ****p < 0.0001.

Initial functional screening was performed using a YFP-based anion influx assay (Figure 3). Extracellular chloride was replaced by iodide because iodide is a much more effective quencher of YFP fluorescence (Kruger et al., 2005). Although the relative permeability of iodide is about 2.3-fold greater than that of chloride (Fatima-Shad and Barry, 1993), the single channel conductance of GlyRs is not significantly changed when chloride is replaced by iodide (Bormann et al., 1987). As all the mutations investigated in this study are at a considerable distance from the ion selectivity filter (Keramidas et al., 2004) it is reasonable to assume that any difference in relative anion permeability will not have any impact on our results. Unless otherwise indicated, a saturating glycine concentration (10 mM) was used in all experiments. Figure 1 shows sample images of HEK293 cells expressing YFP plus the indicated GlyR constructs, taken before and after the addition of 10 mM glycine. These experiments confirmed that the wild-type human α4 GlyR, when recombinantly expressed in HEK293 cells, does not form functional channels (Figures 3A,B). Restoration of the arginine residue at X390 in two independent constructs (X390R I and II) was insufficient to restore human GlyR α4 subunit function. By contrast, expression of artificially synthesized gorilla and chimp GlyR α4 subunit cDNAs generated robust glycine-gated anion influxes (Figures 3A,B), confirming that the GlyR α4 subunit is functional in two species closely related to humans. Taken together, these data suggest that the human GlyR α4 subunit gene harbors further damaging changes compared to functional mouse, gorilla and chimp GlyR α4 subunits. Outside the variable M3-M4 loops, key differences between the human GlyR α4 subunit and rat/mouse sequences include: (1) Signal peptide: E3D, T8L/T8P; (2) Extracellular domain: S55G, I57V, K59E, S80A, L145P, K170N, V174M, C204Y; (3) Transmembrane domain M3: I311V; and (4) C-terminal extracellular loop: W421R (Figure 2). Again, the X390 stop codon is not present in either mouse or rat GlyR α4 subunits, where an arginine is present.

We assessed the potentially damaging effects of these changes in the GlyR α4 subunit using software packages SIFT and PolyPhen-2. SIFT prediction is based on the degree of conservation of amino acid residues in sequence alignments derived from closely related sequences, collected through PSI-BLAST (Kumar et al., 2009). PolyPhen-2 (Polymorphism Phenotyping v2) is a tool that predicts possible impact of an amino acid substitution on the structure and function of a given protein using straightforward physical and comparative considerations (Adzhubei et al., 2013). We used the mouse GlyR α4 subunit as a starting point, since this subunit is known to be functional in electrophysiological assays (Harvey et al., 2000). The results are summarized in Table 1. While most substitutions were tolerated/benign, E59K and R421W were predicted to be *not tolerated* by SIFT, and E59K, P145L, Y204C, V311I and R421W were predicted to be *possibly or probably damaging* by PolyPhen-2. E59K results in a change from a negatively-charged residue (E, glutamate) to a positively-charged residue (K, lysine). R421W results in a change from a positively-charged residue (R, arginine) to a large aromatic residue (W, tryptophan). However, it is noteworthy that most of the amino acids that are predicted to be damaging by SIFT and PolyPhen-2 (K59, L145, I311, W421) are unique to the human GlyR α4 subunit (Figures 1, 2). The exception is C204, which introduces an additional reactive cysteine into the ECD. GlyRs typically have five cysteine residues in the ECD, four of which form disulfide bonds that are important for cell-surface expression and ECD folding (Vogel et al., 2009). In the GlyR α1 subunit, these form a signature disulfide loop (Cys^138^-Cys^152^) that is also found in nAChRs, 5HT_3_Rs and GABA_A_Rs. In addition, a second GlyR-specific disulfide bond is formed (Cys^198^-Cys^209^), leaving one cysteine (Cys^41^) unpaired (Vogel et al., 2009). The equivalent residues in the human GlyR α4 subunit are Cys^144^-Cys^158^, Cys^205^-Cys^216^ and Cys^47^. However, the human, chimp and gorilla GlyR α4 subunits all contain an extra cysteine (Cys^204^) that may have a significant impact on disulfide bond formation (Figure 1).

**Table 1:** Prediction of potentially damaging changes in the human Inhibitory glycine receptor (GlyR) α4 subunit compared to the mouse GlyR α4 subunit.

Change	SIFT	PolyPhen-2
D3E	Tolerated	Benign (score 0)
L8T	Tolerated	Benign (score 0.002)
G55S	Tolerated	Benign (score 0.003)
E59K	Not tolerated (change in charge)	Probably damaging (0.972)
A80S	Tolerated	Benign (score 0.046)
P145L	Tolerated	Possibly damaging (0.951)
N170K	Tolerated	Benign (score of 0.044)
M173V	Tolerated	Benign (score of 0)
Y204C	Tolerated	Probably damaging (0.993)
V311I	Tolerated	Possibly damaging (0.924)
M322I	Tolerated	Benign (score of 0)
R339Q	Tolerated	Benign (score of 0.01)
S366G	Tolerated	Benign (score of 0)
T374A	Tolerated	Benign (score of 0)
K378R	Tolerated	Benign (score of 0)
M383T	Tolerated	Benign (score of 0)
R390X	-	-
V408I	Tolerated	Benign (score of 0)
R421W	Not tolerated (charge—bulky residue)	Probably damaging (1)

*Individual amino acid substitutions were made in the mouse GlyR α4 subunit (Figure 2; Harvey et al., 2000) and potentially damaging effects were assessed using SIFT (http://sift.jcvi.org/) and PolyPhen-2 (http://genetics.bwh.harvard.edu/pph2/)*.

To test the impact of the potentially damaging residues in the human GlyR α4, we introduced G55S, E59K, P145L, Y204C, V311I and R421W changes into the functional mouse GlyR α4 subunit (Harvey et al., 2000). Upon functional expression in HEK293 cells, we found that the E59K substitution completely precluded functional expression, whereas the Y204C change significantly reduced the maximal change in fluorescence, suggesting either impaired receptor trafficking, and/or receptor function (Figure 3C). The other mutations had no deleterious effect (Figure 3C). To test whether human GlyR α4 function could be restored by reversing these mutations, residues in human GlyR α4 were changed to the equivalent residues present in mouse GlyR α4. Since E59K precluded functional expression in mouse GlyR α4, we made the K89E change alone and together with other potentially damaging mutations, K59E, K59E/C204Y, K59E/W421R and K59E/C204Y/W421R, in both the truncated (X390) and restored (R390) human GlyR α4 subunit. As shown in Figure 3D, GlyR function could not be restored by introducing K59E alone in either the GlyR α4^X390^ or α4^R390^ backgrounds. However, introduction of K59E with either C204Y or W421R restored function in the GlyR α4^R390^ background. This strongly suggests that multiple damaging changes—in particular K59E and X390R, but also C204Y and W421R—completely preclude function of the human GlyR α4 subunit *in vivo*.

Since GlyRs truncated in the M3-M4 loop can be incorporated into functional GlyRs when co-expressed with wild-type GlyR subunits (e.g., the hyperekplexia mutation p.E375X, Bode et al., 2013), we tested whether wild-type human GlyR α4 subunits could act as negative regulators of other GlyR subtypes, as proposed by Labonne et al. (2016). While wild-type homomeric GlyR α1 and heteromeric α1β subunit GlyRs generated robust glycine-induced fluorescence responses (Figure 3E), the wild-type human GlyR α4 subunit harboring the X390 truncating mutation (GlyR α4^X390^) was incapable of forming functional homomeric or heteromeric GlyRs (Figures 3B,E). Co-expression of human GlyR α1 and α4^X390^, or α1, α4^X390^ and β subunits did not result in significant changes in maximal fluorescence quench, suggesting that co-expression of the native human GlyR α4^X390^ does not influence function of homomeric α1 or heteromeric α1β GlyRs (Figure 3E). An artificial mutant GlyR α4^X390^ subunit that also incorporated the K59E and C204Y mutations was also incapable of forming functional homomeric or heteromeric GlyRs, but did reduce the observed maximal fluorescence quench for when co-expressed with GlyR α1 or α1β GlyRs (Figure 3E), suggesting that the artificial mutant GlyR α4^K59E/C204Y/X390^ was incorporated into functional pentamers.

**Figure 4:**
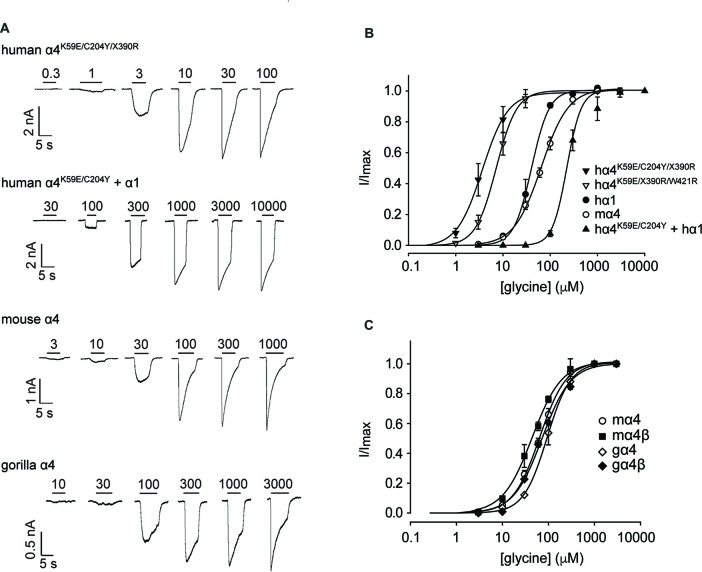
Functional analysis of human, mouse and gorilla α4 subunit GlyRs using whole-cell patch-clamp electrophysiology. **(A)** Glycine dose-response sample traces for truncated and full-length human α4^K59E/C204Y^ GlyRs. The truncated form was co-expressed with the wild-type GlyR α1 subunit. Glycine dose-response sample traces for wild-type mouse and gorilla α4 GlyRs are also shown. Filled bars indicate the applied glycine concentration in micromolar. **(B)** Normalized, averaged glycine dose-response results for the indicated human GlyRs, together with wild-type mouse α4 subunit GlyRs for comparison. Parameters of best fit to the Hill equation are summarized in Table 2. **(C)** Normalized, averaged glycine dose-response results for the indicated mouse and gorilla α4 subunit GlyRs. Parameters of best fit to the Hill equation are summarized in Table 2.

We next sought to confirm the key results from Figure 3 via whole-cell electrophysiology in HEK293 cells. Examples of current traces recorded in response to increasing glycine concentrations for a subset of the investigated constructs are shown in Figure 4A, with averaged concentration-response relationships for all investigated constructs presented in Figures 4B,C. First, glycine concentration-response relations were determined for human α1 and mouse α4 GlyRs (Figure 4B, Table 2). Their respective EC_50_ values were not significantly different from each other. We next characterized the human GlyR α4^K59E/C204Y/X390R^, α4^K59E/X390R/W421R^ and α4^K59E/C204Y/X390^ mutants. The glycine EC_50_ values for the human α4^K59E/C204Y/X390R^ and α4^K59E/ X390R/W421R^ GlyRs were significantly reduced relative to those recorded for the human α1 and mouse α4 GlyRs (Figures 4A,B, Table 2). Although the truncated mutant α4^K59E/C204Y/X390^ GlyR was non-functional when expressed alone, co-expression with the wild-type human GlyR α1 subunit resulted in a significant increase in glycine EC_50_ (from 64 ± 8 to 350 ± 29 μM, *p* < 0.001; Figures 4A,B, Table 2) suggesting that the truncated α4^K59E/C204Y/X390^ mutant can co-assemble with human GlyR α1. However, restoration of two non-conserved residues, E59K and Y204C, was required for co-assembly and it is important to note that this artificial mutant does not exist *in vivo*. Taken together, these data indicate that the wild-type human α4^X390^ GlyR does not contribute to functional GlyRs and cannot act as a negative regulator of other GlyR subtypes.

**Table 2:** Properties of GlyRs measured using whole-cell patch-clamp electrophysiology.

GlyR construct	EC_50_ (μM)	*n*_H_	*I*_max_ (nA)	*n*
Human α1	41 ± 6	2.7 ± 0.3	2.9 ± 0.3	6
Human α4^K59E/X390R/W421R^	7.6 ± 1.3*	2.2 ± 0.1	2.3 ± 0.5	5
Human α4^K59E/C204Y/X390R^	4.3 ± 1.3*	2.1 ± 0.3	2.8 ± 0.5	5
Human α4^K59E/C204Y^ + α1	350 ± 29***	3.1 ± 0.4	3.9 ± 0.6	4
Mouse α4	61 ± 10	1.6 ± 0.1	2.8 ± 0.6	9
Mouse α4β	45 ± 13	1.8 ± 0.1	3.8 ± 0.5	8
Gorilla α4	86 ± 16	1.8 ± 0.1	1.7 ± 0.7	5
Gorilla α4β	71 ± 13	1.7 ± 0.2	0.9 ± 0.2	9

EC_50_ values, Hill coefficients (n_H_) and the maximal currents (I_max_) are represented. p-values were calculated relative to α1 subunit GlyRs using one-way ANOVA followed by Dunnett’s post hoc test: *p < 0.05, ***p < 0.0001.

Figure 3A also suggests that the gorilla and chimp α4 GlyRs express strongly, despite incorporating Cys^204^ residues that contribute to the impairment of the functional expression of human α4 GlyRs. To confirm this, we quantified the glycine concentration-response relationships of the mouse α4 and α4β GlyRs and the gorilla α4 and α4β GlyRs. When expressing heteromeric GlyRs, we transfected α4 and β subunits at a ratio of 1:100 given that has been shown to maximize the expression of heteromeric GlyRs in HEK293 cells (Zhang Y. et al., 2015). Examples of currents recorded in response to increasing glycine concentration for the mouse and gorilla homomeric GlyRs are shown in Figure 4A, with averaged results for homomeric and heteromeric GlyRs presented in Figure 4C and Table 2. There was no significant difference in EC_50_ values among the four receptors, indicating no deleterious effect of the gorilla α4 GlyR Cys^204^ residue.

### Properties of IPSCs Mediated by Mouse and Gorilla α4β GlyRs in Artificial Synapses

**Figure 5:**
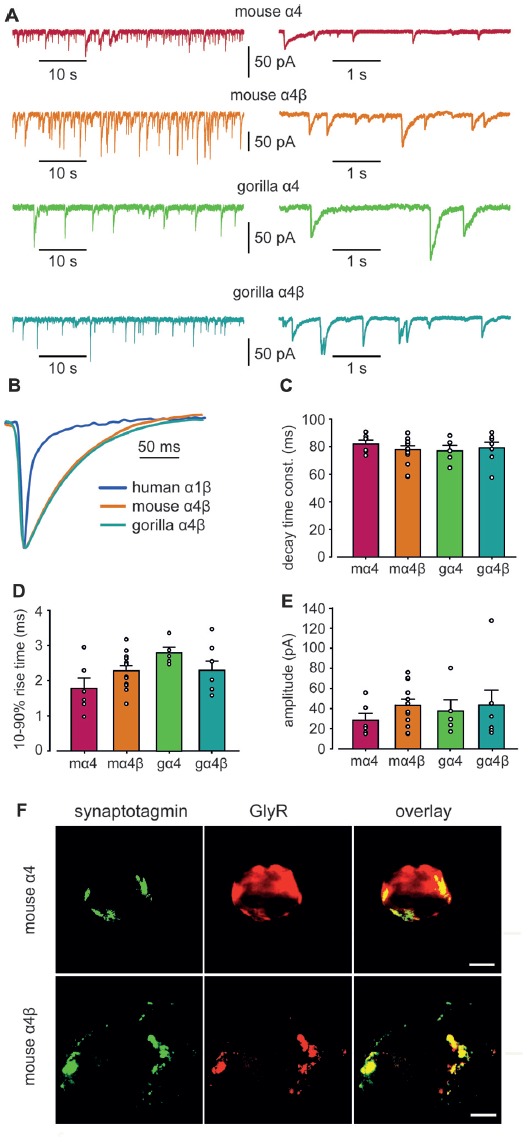
Properties of spontaneous inhibitory postsynaptic currents (IPSCs) recorded from artificial synapses incorporating mouse α4, mouse α4β, gorilla α4 and gorilla α4β GlyRs. **(A)** Representative recordings of IPSCs from HEK293 cells expressing each isoform at two temporal scales. **(B)** Averaged (from 20 to 100 events), normalized IPSCs from individual cells expressing mouse α4 and α4β GlyRs. A corresponding waveform from a human α1β GlyR is also shown. **(C–E)** Mean IPSC decay time constants, 10%–90% rise times and amplitudes. *n* values ranged from 5 to 12. Means were tested for significance using one-way ANOVA although no statistically significant differences were found. **(F)** Images of HEK293 cells that receive artificial synaptic contacts from co-cultured spinal neurons. Images in the top and bottom rows are from the same cells that were transfected with mouse α4 and α4β GlyRs, respectively. Immunolabeling for GlyR (green), synaptotagmin (red) and overlay (yellow) are shown for each cell.Immunolabeling for synaptotagmin (green), GlyR (red) and overlay (yellow) are shown for each cell. Scale bars = 5 μm.

Whole-cell recordings from transfected HEK293 cells in co-culture with spinal neurons regularly exhibited robust, spontaneous IPSCs mediated by mouse α4 and α4β GlyRs and gorilla α4 and α4β GlyRs (Figure 5A). Figure 5B shows digitally averaged and normalized IPSCs from single HEK293 cells expressing mouse and gorilla α4β GlyRs, together with a control IPSC mediated by α1β GlyRs. Mean IPSC 10%–90% rise times, decay time constants and amplitudes, presented in Figures 5C–E, reveal no significant differences among the four α4-subunit containing GlyRs. However, it is evident from Figure 5B that IPSCs mediated by α4β GlyRs exhibit dramatically slower decays than those mediated by α1β GlyRs. Indeed, the mean decay time constant of IPSCs mediated by α4-containing GlyRs (~80 ms; Figure 5C) is drastically slower than those mediated by either α1β, α2β or α3β GlyRs (7.2, 25.7 and 9.7 ms, respectively; Zhang Y. et al., 2015).

To investigate the mechanism responsible for the slow IPSC decay, we first sought to determine whether α4β GlyRs were located in clusters apposed to presynaptic terminals in artificial synapses. We employed immunofluorescence to compare the degree to which mouse α4 and α4β GlyRs co-localized with synaptotagmin, a presynaptic marker. Sample images of single co-cultured HEK293 cells transfected with α4 and α4β GlyRs, respectively, are shown in Figure 5F. The percentage overlap between synaptotagmin and GlyR immunofluorescence was 36 ± 13% (*n* = 4 cells) for α4 GlyRs and 69 ± 5% (*n* = 7 cells) for α4β GlyRs. The difference was significant (*p* < 0.02 by ANOVA), indicating that α4β GlyRs exhibit more pronounced clustering. This is expected given that β subunits mediate GlyR anchoring to the synapse via a direct interaction with gephyrin (Meyer et al., 1995), which are both recombinantly expressed in our artificial synapse system.

**Figure 6:**
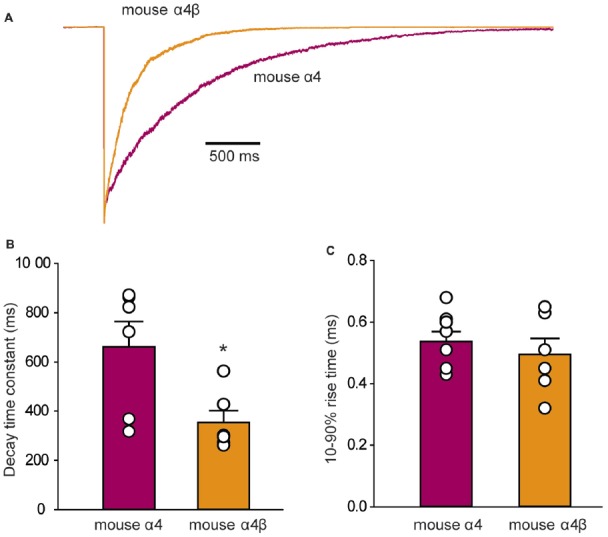
Outside-out macropatch recordings of currents mediated by mouse α4 and α4β GlyRs. **(A)** Sample recordings from macropatches expressing the indicated isoforms in response to ≤1 ms applications of saturating (3 mM) glycine. Patches were clamped at −70 mV and 15–40 sweeps were averaged from each recorded patch. **(B)** Mean deactivation time constants reveal a significant difference between the two isoforms (*p = 0.02, *n* = 6 patches for both). **(C)** Mean 10%–90% activation times reveal no significant difference between isoforms.

To determine whether the IPSC decay rate was determined by the intrinsic receptor closing rate, we recorded ensemble currents from outside-out patches excised from HEK293 cells that expressed either mouse α4 or α4β GlyRs. To mimic synaptic activation conditions, we applied a saturating (3 mM) glycine concentration for 1 ms via a piezoelectric translation device. Examples of ensemble currents activated under these conditions for the two isoforms are shown in Figure 6A with the mean deactivation time constants and 10%–90% rise times (averaged from 6 patches each) summarized in Figures 6B,C. A two-way ANOVA revealed no statistically significant difference in rise times. However, the mean decay time constant for α4 GlyRs (661 ± 103 ms) was significantly slower than that for α4β GlyRs (354 ± 48 ms; *p* < 0.05 by ANOVA). These decay times are substantially slower than those for α1 and α1β GlyRs recorded under similar conditions. Homomeric α1 subunit GlyRs decay with a time constant of 24 ms (Scott et al., 2015), whereas heteromeric α1β subunit GlyRs decay with time constants that range from 16 ms (Scott et al., 2015) to 26 ms (Zhang et al., 2016). Thus, the deactivation time constant for the α4β GlyR is an order of magnitude slower than for the α1β GlyR. Moreover, we observed that, as for α1 subunit GlyRs, currents mediated by α4-containing GlyRs exhibit faster decay times when expressed at synapses. For instance, α1β and α4β GlyR currents decay ~3–4-fold faster at synapses compared to macropatch recordings, likely reflecting the presence of intracellular modulatory factors that shape IPSCs that are removed upon macropatch excision.

### A Zebrafish Gene Trap Line Reveals *glra4a* Expression in Brainstem and Spinal Cord Neurones

**Figure 7:**
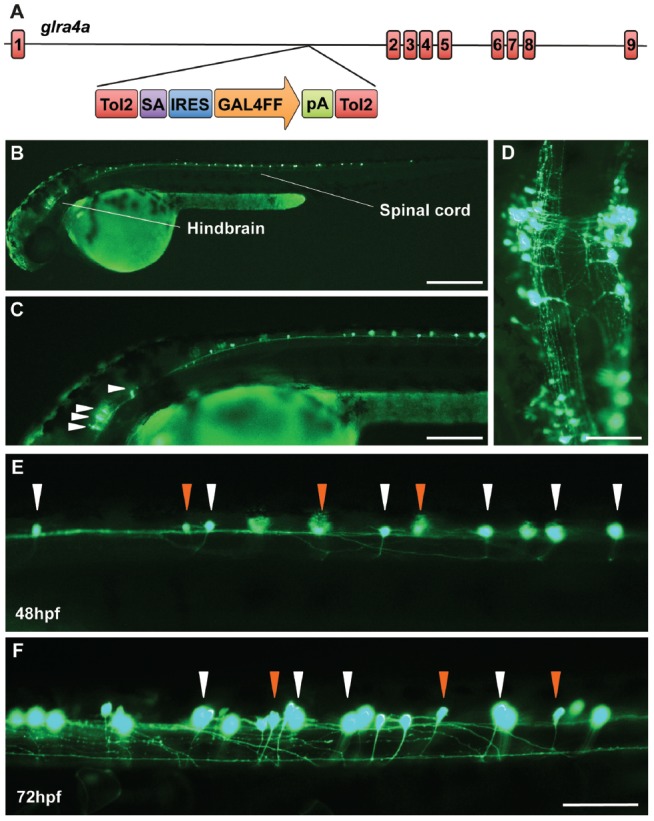
A novel zebrafish *glra4a* gene trap reveals expression in the zebrafish brain and spinal cord. **(A)** The zebrafish SAIGFF16B line contains the gene trap construct T2KSAGFF inserted between exons 1 and 2 of *glra4a*. This cassette contains a splice acceptor (SA), followed by an internal ribosome entry site (IRES), the coding region for the Gal4FF transcription activator and a polyadenylation site (pA). Gal4FF expression in SAIGFF16B was visualized by creating double transgenic fish carrying the Gal4FF transgene and a GFP reporter gene placed downstream of the Gal4-recognition sequence (UAS:GFP). **(B,C)** GFP expression in the *glra4a* gene-trap line at 48 hours post fertilization (hpf) reveals that *glra4a* is predominantly expressed four clusters of hindbrain commissural neurons and selected spinal commissural interneurons. **(D)** High magnification dorsal view of neurons indicated by arrows in **(C)**. **(E,F)** Images show a portion of the zebrafish spinal cord at 48 hpf **(E)** and 72 hpf **(F)** showing *glra4a* expression in commissural primary (CoPA, white arrows) and secondary (CoSA, orange arrows). Scale bars: **(B)**: 500 μm; **(C)**; 250 μm; **(D)**: 150 μm; **(E,F)**: 50 μm.

In order to learn more about the biological role of GlyR α4 we turned to zebrafish, which have two GlyR α4 subunit genes (*glra4a* and *glra4b*) with distinct expression patterns (Imboden et al., 2001; Hirata et al., 2010; Hensley et al., 2011). In 24 hpf embryos, *glra4b* (previously known as αZ4) expression was previously reported to be restricted to the rhombencephalic portion of the midbrain-hindbrain boundary and the rhombic lip, but from 52 hpf was confined to the retina (Imboden et al., 2001; Hensley et al., 2011). By contrast, *glra4a* (previously known as αZ2) was reported to be more widely expressed in the olfactory pits, the mesencephalon, the rhombencephalon and the somites (Imboden et al., 2001). In this study, we used a novel Tol2 based gene-trap line (Kawakami et al., 2010), to map the expression of *glra4a* in more detail. The zebrafish line SAIGFF16B contains a reporter cassette integrated into *glra4a* between exon 1 and exon 2. This cassette encodes the yeast transcription factor activator protein GAL4, flanked by IRES and a polyadenylation signal (Figure 7A). GAL4 works efficiently as a transcription factor when it binds to an upstream activator sequence (UAS) located near a fluorescent protein such as enhanced green fluorescent protein (EGFP). The zebrafish *glra4a*:GAL4 gene-trap line was therefore crossed with a UAS:EGFP line. The offspring express GAL4, which binds to the UAS and subsequently triggers the production of EGFP. Therefore, areas within the zebrafish where EGFP is detected indicate cells where *glra4a*:GAL4 is normally expressed. EGFP expression directed by *glra4a*:GAL4 at 48 hpf revealed that *glra4a* was predominantly expressed four clusters of hindbrain commissural neurons and selected spinal commissural interneurons (Figures 7B–D). The expression of *glra4a*:GAL4 in commissural primary and secondary ascending neurons of the spinal cord increased in intensity from 48 hpf to 72 hpf (Figures 7E,F). These spinal commissural neurones had two distinct morphologies: large soma with multidendritic processes and small soma with few dendrites. Interestingly, no motor neurons or other types of interneurons were labeled in this line.

### Morpholino Knockdown and Overexpression of an Artificial GlyR α4a R278Q Mutation Reveals Aberrant Swimming Behavior

**Figure 8:**
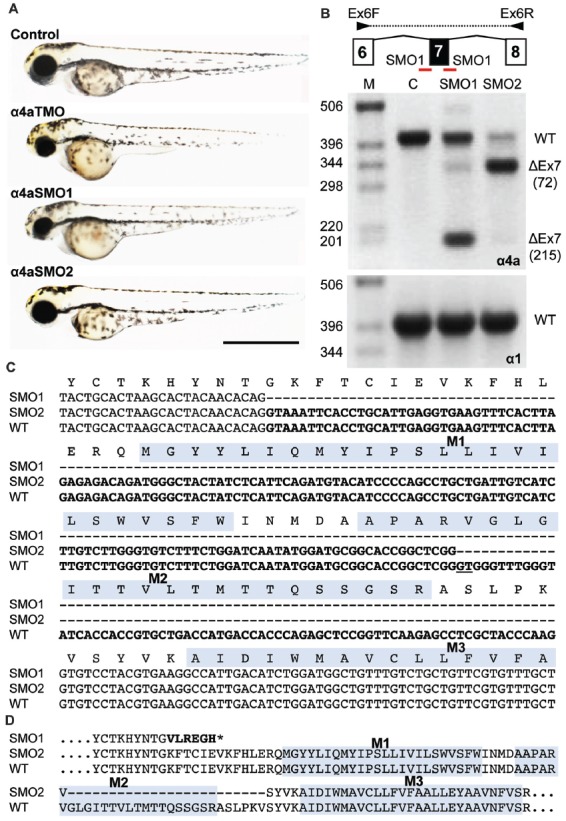
Morpholino oligonucleotides (SMO1 and SMO2) block splicing of *glra4a*. **(A)** Gross morphology of wild-type embryos and those injected with GlyR α4a translation-blocking (TMO) or splice site (SMO1 and SMO2) morpholinos. **(B)** Schematic of RT-PCR analysis of *glra4a* morpholinos confirmed the deletion of exon 7 for α4a-SMO1 (lane 3) and the deletion of part of exon 7 for α4a-SMO2 (lane 4). Intact GlyR α4a exon 7–9 PCR products are seen in the control (393 bp, lane 2) and smaller amounts are also observed in lanes 3 and 4. A fragment of around 178 bp is seen in SMO1, which is made up of exon 6 and 8 only. A fragment of 321 bp in SMO2 contains exons 6 and 8 and part of exon 7, which excludes the region that codes for TM2. GlyR α1 exon 7 is present in control and both SMO fish (lower panel). **(C,D)** DNA sequencing of GlyR α4a cDNAs from SMO1 and SMO2 treated fish reveals that SMO1 results in a 215 bp deletion, resulting in a frameshift and premature stop codon before M1, while SMO2 results in a 72 bp deletion and loss of 24 amino acids, including the majority of the pore-forming M2 domain.

Splice site (SMO) and translation blocking (TMO) morpholinos α4a-SMO1, α4a-SMO2 and α4a-TMO were injected into zebrafish embryos. The morphology of the injected zebrafish was assessed at 48 hpf (Figure 8A) and did not reveal any gross anatomical changes apart from slightly smaller eyes that were apparent in embryos injected with α4a-TMO and α4a-SMO1 (Figure 8A). Although it was not possible to monitor the efficacy of α4a-TMO knockdown, due to the lack of a specific GlyR α4a subunit antibody, RT-PCR was used to monitor the effects of α4a-SMO1 and α4a-SMO2. mRNA was extracted from zebrafish that had been injected with α4a-SMO1 and α4a-SMO2 plus non-injected zebrafish embryos (wild-type control). RT-PCR was performed using primers targeted within exon 6 (forward) and exon 8 (reverse) of zebrafish *glra4a* and the PCR products corresponding to exons 6–8 analyzed by agarose gel electrophoresis and DNA sequencing (Figure 8B). This analysis suggested that binding of α4a-SMO1 to the splice acceptor site resulted in skipping of exon 7 in a proportion of transcripts (note lower 178 bp band), whilst binding of α4a-SMO2 to the exon 7 acceptor site resulted in mis-splicing, so that a different “acceptor” site was used within exon 7. DNA sequencing of GlyR α4a cDNAs from SMO1 and SMO2 treated fish revealed that SMO1 indeed resulted in exon 7 skipping (a 215 bp deletion) resulting in a frameshift and premature stop codon before M1. SMO2 results in usage of an exonic donor site, resulting in a 72 bp deletion and loss of 24 amino acids, including the majority of the pore-forming M2 domain (Figure 8C). By contrast, RT-PCR with similar primers targeted against zebrafish *glra1* did not result in any aberrant splicing (Figure 8B, lower right panel) suggesting that the GlyR α4a morpholinos used did not recognize this closely related target.

**Figure 9:**
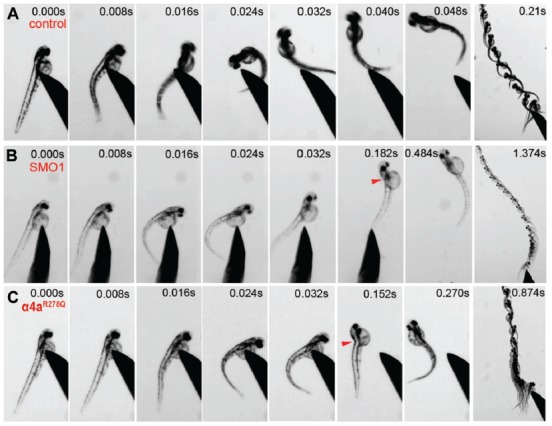
Embryos injected with α4a-SMO1 and the α4a^R278Q^ mutant embryos fail to exhibit correct escape behavior. **(A)** Wild-type (control) fish responds to touch by with a C-bend, a turn away from the stimulus and rapid swimming. However, embryos injected with α4a-SMO1 **(B)** or with mRNA for the GlyR α4a^R278Q^ subunit **(C)** respond more slowly, and have a pronounced head retraction—similar to a localized *beo*-like contraction (arrow). Single images were extracted from high-speed movies of α4a-SMO1 morphants and embryos injected with GlyR α4a^R278Q^ subunit mRNA.

Wild-type zebrafish embryos respond to tactile stimulus with an “escape response” consisting of a C-bend, a counter-turn, and a bout of rapid swimming (Figure 9A). However, behavioral analysis of zebrafish embryos injected with α4a-SMO1 gave rise to transient spasms and prolonged head retraction (Figure 9B). α4a-SMO1 morphants did eventually recover and swim away from the stimulus, although this took longer than for wild-type zebrafish (Figures 9A,B). Thus, knockdown with α4a-SMO1 significantly impaired escape behavior. The same behavior was observed in embryos injected with *in vitro*-transcribed RNA for a GlyR α4a subunit mutant harboring the R278Q mutation (Figure 9C; GlyR α4a^R278Q^). This mutation is equivalent to the dominantly inherited startle disease mutation p.R271Q in the human GlyR α1 subunit (Shiang et al., 1993) that disrupts the link between agonist binding and channel gating without affecting cell-surface trafficking (Langosch et al., 1994; Chung et al., 2010). This suggests that the GlyR α4a^R278Q^ dominant-negative mutant is able to incorporate into native zebrafish GlyRs and alter behavior in the same manner as morpholino knockdown by α4a-SMO1, resulting in aberrant tactile startle and escape responses.

## Discussion

The known biological roles of inhibitory GlyRs have expanded significantly in recent years, in part due to the range of mouse/zebrafish mutants and subtype-specific antibodies now available (Harvey et al., 2004; Hirata et al., 2010; Pilorge et al., 2016; Wilkins et al., 2016; Schaefer et al., 2017). However, GlyRs containing the α4 subunit have largely escaped scrutiny, largely because the human gene has an in-frame stop codon in exon 9, truncating the GlyR α4 subunit in the M4 domain. The GlyR α4 subunit gene is clearly intact and expressed in other organisms (e.g., Matzenbach et al., 1994; Harvey et al., 2000) and is even duplicated (GlyR α4a and α4b) in zebrafish (Imboden et al., 2001; Hirata et al., 2010). Given the recent implication of the human GlyR α4 subunit gene in human disease, we sought to: (1) Determine whether the human GlyR α4 subunit is indeed functional or can act in a dominant-negative manner; (2) To characterize the functional properties of α4-subunit GlyRs in artificial synapses; and (3) Determine the biological role of GlyR α4 using zebrafish as a model organism.

### The Human GlyR α4 Subunit Is Functionally Inactive Due to Multiple Damaging Changes

Cloning and sequencing of human GlyR α4 subunit cDNAs (Figures 1, 2) demonstrated that *GLRA4* is still transcribed and correctly spliced in human brain. However, no alternative splicing or RNA editing was observed that could “correct” the stop codon found at position 390. Functional expression of “wild-type” human GlyR α4^X390^ did not result in functional GlyRs, although artificially-synthesized gorilla and chimpanzee α4 subunit cDNAs could direct the formation of functional GlyRs (Figure 3A). It was therefore surprising that mutagenesis of the stop codon in human GlyR α4 to a conserved arginine (p.X390R) did not restore function. Bioinformatics analysis using SIFT and PolyPhen-2 and further mutagenesis of the mouse and human GlyR α4 subunit cDNAs (Figures 3B,C) revealed that the human GlyR α4 subunit contains *multiple damaging changes*—including K59E and X390R, and to a lesser extent C204Y and W421R—that inactivate human GlyR α4 subunit function. Restoration of multiple amino acids (K59E with either C204Y or W421R) was required to restore function in the GlyR α4^R390^ background. It is curious that either C204Y or W421R had an equivalent effect in restoring function given that: (1) these residues are not located near each other; and (2) incorporation of either residue together with K59E and X390R results in α4 GlyRs with very high glycine sensitivity (EC_50_s: α4^K59E/X390R/W421R^: 7.6 ± 1.3 μM; α4^K59E/C204Y/X390R^: 4.3 ± 1.3 μM; Table 2). The similarly high glycine sensitivities of the human α4^K59E/C204Y/X390R^ and α4^K59E/X390R/W421R^ GlyRs are presumably caused by other residues that are not conserved between human and other species (e.g., L145, K170, I311). This finding also indicates that the individual C204 or W421 human residues do not completely impair GlyR function, which is supported by our mouse GlyR α4 mutagenesis study (Figure 3B) and the fact that we observed no deleterious effect of the gorilla GlyR α4 Cys^204^ residue in whole-cell patch-clamp experiments that compared mouse α4 and α4β GlyRs with gorilla α4 and α4β GlyRs (Figure 4). This in turn rules out the possibility that the loss of function in the α4^K59E/X390R^ GlyR is a cumulative effect of the two mildly “deleterious” endogenous residues (i.e., C204 and W421). Lastly, we also found that “wild-type” GlyR α4^X390^ subunit was unable to act as a negative regulator of other GlyR subtypes (Figures 3D, 4). This was at first surprising, given that several elegant studies (Villmann et al., 2009; Haeger et al., 2010) have shown that truncated proteins containing the N-terminal ligand-binding domain and first three transmembrane helices (M1–M3) of the GlyR α1 subunit can be rescued by co-expressing the fourth transmembrane domain (M4). However, additional artificial restoration of K59E/C204Y was required before co-assembly with the GlyR α1 subunit was observed. Hence, we consider it highly unlikely that the endogenous GlyR α4 subunit is involved in human disease by the mechanisms proposed by Labonne et al. (2016). Rather, sequence analysis of high coverage reads from the Denisovan genome (Reich et al., 2010) suggest that *GLRA4* has been a pseudogene for at least 30,000–50,000 years.

### Unique Functional Properties of α4 Subunit GlyRs Revealed in Artificial Synapses and Outside-Out Patches

Until this study, the functional properties of IPSCs mediated by α4-containing GlyRs had not been determined for any species. We therefore made whole-cell recordings from HEK293 cells expressing mouse or gorilla α4 and α4β GlyRs along with neuroligin 2 that had been co-cultured with spinal neurons. In this system, expression of GlyR α1–3 homomers typically results in robust IPSCs, with GlyR β subunit incorporation accelerating IPSC rise and decay times for α2β and α3β heteromers. In addition, α1β and α3β GlyRs mediate fast decaying IPSCs, whereas α2β GlyRs mediate slow decaying IPSCs (Zhang Y. et al., 2015). In this study, we show that analysis of IPSCs (Figure 5) and analysis of IPSC 10%–90% rise times, decay time constants and amplitudes, revealed no significant differences between homomeric α4 and heteromeric α4β-subunit mouse and gorilla GlyRs (Figure 5). IPSCs mediated by α4 or α4β GlyRs had a dramatically slower mean decay time constant (~80 ms; Figure 5C) than those mediated by either α1β, α2β or α3β GlyRs (7.2, 25.7 and 9.7 ms, respectively; Zhang Y. et al., 2015). Another unusual finding was that the intrinsic closing rate of the mouse α4 and α4β GlyRs in outside-out patches excised from HEK293 cells (Figure 6B) was much slower than the corresponding IPSC decay rate (Figure 5B). One possible explanation is that the synaptic clustering process imposes a conformational change upon α4-containing GlyRs that induces them to close at a faster rate. We were surprised to find that the α4β GlyR deactivation time constant was an order of magnitude slower than for the α1β GlyR (Scott et al., 2015; Zhang et al., 2016). We are not aware of any other pentameric ligand-gated ion channel that deactivates at such a slow rate. Taken together, these findings suggest that α4 subunit-containing GlyRs have a unique physiological function and are possibly more suited to tonic rather than fast synaptic signaling.

### A Biological Role for GlyR α4 in Startle and Escape Responses

In order to develop an *in vivo* model of GlyR α4 dysfunction, we utilized zebrafish, where an extended GlyR gene family (α1, α2, α3, α4a, α4b, βa and βb) presents many advantages for the study of receptor biology (Hirata et al., 2010, 2013; Ganser et al., 2013; Pilorge et al., 2016). Zebrafish also present other benefits in terms of the availability of artificial mutants and techniques for gene manipulation. For example, zebrafish bandoneon (*beo*) mutants harbor missense or nonsense mutations in *glrbb* that cause compromised glycinergic transmission and touch-induced bilateral muscle contractions (Hirata et al., 2005; Ganser et al., 2013). We have also previously demonstrated that GlyR gene-specific morpholinos can reveal distinct phenotypes in zebrafish larvae, such as embryonic spasticity (GlyR α1; Ganser et al., 2013) and axon-branching defects (GlyR α2; Pilorge et al., 2016). Zebrafish contain two GlyR α4 subunit genes, *glra4a* and *glra4b*, with the expression of the latter gene being largely restricted to the retina (Imboden et al., 2001; Hensley et al., 2011). Using a novel Tol2-based gene-trap line, we detected *glra4a* expression in small interneuron populations in the hindbrain and commissural primary and secondary ascending neurons of the spinal cord (Figure 7). We tested and validated multiple splice-site and translation blocking morpholinos (MOs) targeting *glra4a* using RT-PCR and DNA sequencing to show exon skipping and alternate acceptor site usage for exon 7 MOs resulting in truncation of zebrafish GlyR α4a (Figure 8). High-speed video analysis of zebrafish embryos injected with α4a splice site morpholino (SMO1) or a dominant-negative GlyR α4a^R278Q^ subunit mutant gave rise to transient spasms and prolonged head retraction after a tactile stimulus (Figure 9). Thus, *glra4a* knockdown in zebrafish results in aberrant tactile escape responses, suggesting that at least in fish, GlyR α4a helps to shape startle and escape responses. Given the known role of the spinal GlyR α1β subtype in startle disease in humans and animal models (Shiang et al., 1993; Ganser et al., 2013; Wilkins et al., 2016; Schaefer et al., 2017), a role for GlyR α4 in escape behaviors is not unexpected. However, one obvious area for future study is whether loss of GlyR α4 in humans has resulted in key differences in terms of startle and/or escape responses compared to other species.

## Author Contributions

RJH, MS and JWL designed the experiments. KK generated the *glra4a* gene trap line. SL, PS, AB, VMJ, AK, MS, JWL and RJH performed the experiments. SL, PS, AB, VMJ, AK, JWL and RJH analyzed the data. RJH and JWL wrote the article. All authors were involved in revising the article for important intellectual content, and gave final approval of the version to be published.

## Conflict of Interest Statement

The authors declare that the research was conducted in the absence of any commercial or financial relationships that could be construed as a potential conflict of interest.

## Footnotes

^1^http://kawakami.lab.nig.ac.jp/ztrap
